# Potential of Proteomics in Forensic Phenotyping: A Focus on Biological Sex Estimation

**DOI:** 10.1021/acs.jproteome.5c00098

**Published:** 2025-09-03

**Authors:** Shirin Alex, Ruben Almey, Rachel Sian Dennis, Olivier Tytgat, Robbin Bouwmeester, Dieter Deforce, Marcel De Puit, Maarten Dhaenens, Laura De Clerck

**Affiliations:** † Netherlands Forensic Institute, Laan van Ypenburg 6, Den Haag 2497GB, the Netherlands; ‡ ProGenTomics, Faculty of Pharmaceutical Sciences, 26656 Ghent University, Ottergemsesteenweg 460, Ghent 9000, Belgium; § 360916 VIB-UGhent Center for Medical Biotechnology, Technologiepark-Zwijnaarde 75, Ghent 9052, Belgium; ∥ Department of Biomolecular Medicine, Ghent University, Corneel Heymanslaan 10, Ghent 9000, Belgium; ⊥ Faculty of Applied Sciences, Department of Chemical Engineering, Delft University of Technology, Van der Maasweg 9, Delft 2629 HZ, the Netherlands

**Keywords:** forensic phenotyping, proteomics, mass spectrometry, machine learning, sex estimation

## Abstract

Forensic DNA analysis is well established for phenotyping, providing valuable investigative leads. Proteomics, the large-scale study of proteins, is emerging as a complementary tool to DNA analysis, particularly for enhancing the evidential value of traces. This study explores the potential of proteomics to extract phenotypic traits from whole blood, using the estimation of biological sex as a starting point. Using LC–MS/MS, proteomes from 100 whole blood samples of known sex were analyzed to develop a biological sex classifier. Cross-validation of the model highlighted the model’s ability to achieve accurate classification, identifying key peptides, such as those from pregnancy zone protein and ceruloplasmin, as critical markers. To test real-world applicability, mock case samples were generated, bringing attention to the need for model robustness. Overall, our results suggest that using proteomics to infer phenotypic traits from whole blood samples in the context of forensics, while feasible, is hindered by hard-to-overcome technical challenges. We therefore recommend that future forensic proteomics research be directed toward areas where it can be most informative, such as source attribution and estimating the timeline of events, rather than focusing on extracting phenotypic traits for donor profiling.

## Graphical abstract

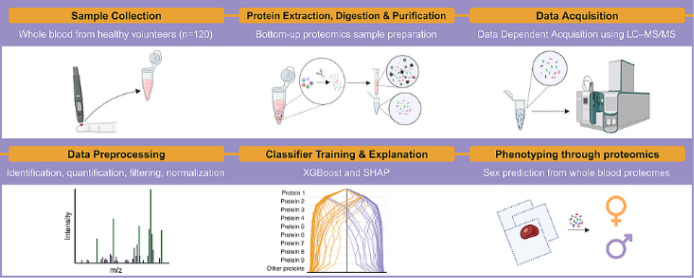

## 1 Introduction

Forensic phenotyping primarily uses deoxyribonucleic acid (DNA) obtained from traces at a crime scene to predict an individual’s external characteristics, age, and biogeographic ancestry.^1^ This allows to profile unknown perpetrators who cannot be identified with the traditional forensic short tandem repeat (STR) analysis.^2^ As a prime example, sex estimation is routinely performed in forensic genetic analysis, especially in cases of sexual assault. Nevertheless, DNA-based predictions of appearance now extend beyond basic features like eye, hair, and skin color, to include traits such as freckles, height, and even male pattern baldness.^3^ The phenotype (i.e., the observable characteristics of an individual) is, however, determined by both genetic makeup and environmental influences, including individual physical, psychosocial, and environmental exposure.^4^ Recent research has demonstrated that the epigenome is highly dynamic, with changes influenced by factors such as nutrition, smoking, alcohol consumption, and drug use.^5^

Advanced analytical techniques, such as liquid chromatography–mass spectrometry (LC–MS), have proven invaluable in forensic investigations, especially in cases involving drug abuse, profiling explosives, and analyzing gunshot residue (GSR), among others.^6^ MS-based proteomics can be particularly useful in cases dealing with heavily degraded samples or when complementary information is needed, thereby enhancing the evidential value of a trace. For instance, analyzing the unique protein compositions of different tissues^7^ and biological fluids,^8,9^ can provide crucial information about the source of a forensic trace. Similarly, understanding post-translational modifications (PTMs), both in vivo and ex-vivo can potentially help provide insights into time since deposition of traces.^10^ Proteomics can also be utilized to obtain valuable information from “challenging” samples, such as hair,^11,12^ and bone,^13,14^ which contain little to no DNA. Over the years, proteomics has become an important forensic research tool^15^ aligning with the field’s goal of maximizing trace-based information.

As proteomics captures information more closely related to the (dynamic) phenotype, we aimed to extract phenotypic traits directly from whole blood samples using LC–MS based proteomics analysis. In this way, proteomics could complement existing DNA-based methods, providing new insights and enhancing forensic analyses. The estimation of biological sex was used as a starting point, it being the most straightforward phenotypic marker to estimate. Currently, estimation of biological sex predominantly relies on typing length variations in the X-Y homologous amelogenin gene (AMELX and AMELY), a method integral to most PCR multiplex kits used in DNA profiling.^16^ MS-based proteomics, on the other hand, can estimate an individual’s sex by analyzing amelogenins in enamel (even from ancient or archeological samples)^17–19^ but also from keratins in hair samples.^20^ However, there are currently no known protein biomarkers for estimating sex from whole blood samples within a forensic context.

Translating protein profiles from whole blood into meaningful phenotypic information is challenged by the highly dynamic nature of the proteome. Additionally, analyzing proteomics data comes with quite a few challenges of its own, such as the complexity of the data characterized by its high dimensionality, various interactions between proteins, and frequent instances of missing data. Machine learning (ML) offers a powerful solution to address these challenges and efficiently manage the complexities.^21,22^ One significant challenge associated with the use of ML pertaining to the forensic field is the need for sufficient training data.^23^ While public data sets can be helpful, there is a notable scarcity of data obtained from whole blood samples even though there is ample clinical serum and plasma data available.

In this exploratory study, we acquire and classify whole blood samples by biological sex through an ML approach, identifying potential biomarker peptides as a proof of concept for forensic proteomics-based phenotyping. Additionally, we address the challenges involved in developing this workflow and what is required before broader adoption of proteomics for forensic phenotyping is possible.

## 2 Methods and Materials

### 2.1 Sample Collection

Prior to sample collection, informed consent was obtained from all volunteers through a detailed informed consent form and after consultation with a privacy officer of The Netherlands Forensic Institute. The form outlined the study’s objectives, the intended use and storage of biological samples and associated data.

Blood samples were collected from 120 healthy volunteers using a single-use lancet (Accu-Chek Safe-T-Pro Plus, Roche, Manheim, Germany) and a 10 μL Mitra microsampling device (Neoteryx, California, USA). To fairly evaluate the machine learning approach, the 120 samples were divided into two subsets: a training set consisting of 100 donors (47 male, 53 female) and a test set consisting of 20 donors (10 male, 10 female). From this point onward, the training and test samples were processed independently.

### 2.2 Protein Extraction, Digestion, and Peptide Purification

After collecting 10 μL of blood, the tip of the single-use sampling device was placed into a Protein LoBind tube (Eppendorf, Hamburg, Germany) containing 590 μL of 50 mM ammonium bicarbonate (ABC) (Sigma-Aldrich, Saint Louis, USA) for whole blood extraction. This was followed by 10 min of sonication to extract both intra- and extracellular proteins and centrifugation at 13,000*g* for 5 min at room temperature.

From the supernatant, 10 μL (∼ 0.17 μL whole blood) was transferred to a Protein LoBind tube (Eppendorf, Hamburg, Germany) followed by the addition of 50 μL of 50 mM ABC (Sigma-Aldrich, Saint Louis, USA). Protein reduction was then performed in 10 mM 1,4-dithiothreitol (DTT) (Roche Diagnostics GmbH, Mannheim, Germany) at 60 °C for 30 min. After incubation, the samples were cooled to room temperature and alkylated in 15 mM methylmethanethiosulfonate (MMTS) (Thermo Fisher Scientific, Waltham, USA), followed by a 10 min incubation in the dark at room temperature. To each sample, 1 μg of MS-grade Trypsin/Lys-C (Promega, Wisconsin, USA) was added, along with 1 mM calcium chloride (CaCl_2_) (Merck, Darmstadt, Germany) and 5% acetonitrile (ACN) (Sigma-Aldrich, Saint Louis, USA) to enhance digestion efficiency. The samples were then incubated overnight at 37 °C. The enzymatic reaction was quenched by adding 10% formic acid (FA) (Sigma-Aldrich, Saint Louis, USA).

Peptide concentration and purification was performed using C-18 SpinTips (Thermo Fisher Scientific, Waltham, USA). The SpinTips were first activated twice with 70% ACN (Sigma-Aldrich, Saint Louis, USA), and centrifuged at 660*g* for 30 s after each step of activation, equilibration, and elution. The tips were then equilibrated three times with 0.1% FA (Sigma-Aldrich, Saint Louis, USA) before loading the digested peptides. After loading, the tips were washed three times with 0.1% FA (Sigma-Aldrich, Saint Louis, USA) and eluted twice with 50 μL of 70% ACN (Sigma-Aldrich, Saint Louis, USA).

### 2.3 Generation of Mock Samples

Mock samples were prepared from the 20 test samples, of which 5 μL of whole blood was deposited onto 1 × 1 cm patches of cotton fabric. These patches were precontaminated with keratins (mainly from skin), dirt, and other plastic contaminants to simulate contamination in real-life forensic samples. After deposition, the samples were left to dry for 1 h at room temperature. The patches were then transferred to clean Protein Lobind tubes (Eppendorf, Hamburg, Germany) containing 1 mL of ultrapure water and incubated for 30 min at room temperature. During this incubation, the samples were vortexed every 10 min to ensure effective extraction of the dried blood from the fabric. After incubation, the samples were centrifuged at 14,000*g* for 5 min and the supernatant was processed using S-Trap spin columns (ProtiFi, New York, USA) according to the manufacturer’s protocol and the eluted peptides were finally vacuum-dried.

### 2.4 Data Acquisition Using LC-MS/MS

The training, test, and mock samples were all acquired using the same instrument but independently and at different time points. The dried peptides were reconstituted in 0.1% formic acid before injecting 400 ng, based on UV/vis quantification using the Lunatic platform (Unchained Laboratories, Pleasanton, USA), onto a Acquity M-class system (Waters, Massachusetts, USA) equipped with a 150 × 0.3 mm Kinetex 2.6 μm XB-C18 column (Phenomenex, California, USA) coupled to a ZenoTOF 7600 (Sciex, Massachusetts, USA) mass spectrometer with the Optiflow TurboV ion source operating in positive mode. All samples were run in fully randomized order to avoid systematic variation with blank runs interspersed to prevent carry-over. Quality controls (QC), composed of a mixture of all samples, were run every 5 samples to monitor instrument performance. Peptides were separated using a 20 min active gradient from 3 to 30% B, with mobile phases A and B consisting of 0.1% FA (Sigma-Aldrich, Saint Louis, USA) in water and 0.1% FA (Sigma-Aldrich, Saint Louis, USA) in ACN (Sigma-Aldrich, Saint Louis, USA), respectively. The column temperature was maintained at 30 °C. Sample acquisition was performed using a top 40 data-dependent acquisition (DDA) scheme with a mass range of *m*/*z* 400–1200 for precursor scans and 140–1750 for fragment scans. Precursors with charges 2 to 4 were selected if they exceeded an intensity threshold of 125 cps, with dynamic exclusion set to 10 s after 2 occurrences. Accumulation times were set to 100 ms for MS1 scans and 11 ms for MS2 scans, resulting in a maximum cycle time of 0.71 s. The ion source operated at 200 °C and 4500 V, with gas pressures set at 20, 55, 45, and 7 psi for gas 1, gas 2, curtain gas, and CAD gas, respectively.

### 2.5 Peptide Identification

Raw data was first converted from the.wiff2 to.mzML format using MSConvert.^24^ The binary encoding precision was set to 32-bit with zlib compression enabled, and the following filters were applied: “peakPicking vendor msLevel = 1-” and “threshold absolute 0 most-intense”. Subsequently, all mzML files were processed together using the Sage search engine (v0.14.7).^25^ The complete configuration is available under the Data availability statement. In brief, the database used was the UniProt human reference proteome,^26^ including only reviewed records and their isoforms (downloaded on 27/03/2024), concatenated with a universal protein contaminant database^26,27^ . Trypsin/P allowing for 1 missed cleavage was specified as the enzyme, with peptide lengths set from 6 to 50 amino acids and masses ranging from 500 to 5000 Da. Cysteine methylthio was set as fixed modification and methionine oxidation and protein N-terminal acetylation were set as variable modifications. The precursor tolerance was set from −500 to 100 Da to perform an open mass search. Finally, label-free quantification was performed using default settings. The label-free quantification output was filtered for spectrum-, peptide-, protein-, and MS1-level q-values ≤ 0.01 before being used as an input for the ML-based analysis.

### 2.6 Data Preprocessing

Failed injections (10 out of the 100 training set samples) caused by either failed sample preparation or instrumental failure were excluded from the data set. Additionally, outlier samples (1 out of the 20 test set samples) were identified and excluded using a batch-sensitized local outlier factor approach. This meant a total of 164 MS runs remained, including training set, test set, mock set, and QC samples. Peptide features were filtered if they originated from contaminant proteins as per the database or if they were inconsistently present in the training set, meaning they were excluded if quantified in less than 75% of the male and female training samples. Tail-robust quantile normalization^28^ was applied to the log_2_-transformed data set to remove technical variations such as loading differences while preserving the signals of consistently high-abundant features like from albumin and hemoglobins and accounting for sample-dependent missing values. The notebook describing preprocessing is available under the code availability statement.

### 2.7 Differential Abundance Analysis

Preprocessed peptide data was summarized to the protein level using the robust summarization function of the QFeatures R package.^29^ MDS plots were constructed from training set protein abundances using the limma R package.^30^ A differential abundance analysis between male and female whole blood samples from the training set was performed using the MSqRob2 R package^31,32^ . A report including all code is available under the code availability statement.

### 2.8 Machine Learning Analysis

A sex classifier was trained on the preprocessed peptide data from the training data set. To estimate the best-case performance, training was first done in a nested cross-validation (CV) loop consisting of two levels: an outer and inner CV loop. The outer CV loop was used to estimate the performance of the fitted model by taking a randomized, stratified, 10-fold sample of 9 instances for testing and 81 for training. These 81 training instances were used to first select the discriminating features after which, inside the inner stratified CV loop comprising 5 splits, the best hyper-parameter set was selected to fit a gradient boosting model (XGBoost v2.1.0.dev0).^33^ A final gradient boosting model was then trained on all available data and explored using Shapley additive explanations (SHAP)^34^ to interpret overall classification decisions and feature contributions to individual predictions. Finally, the predictive performance of the fitted model was evaluated against the test and mock data sets, which were preprocessed in the same manner as the training data set. The full notebook is available under the code availability statement.

## 3 Results and Discussion

### 3.1 The Lack of Whole-Blood Proteomics Data

Most forensic blood traces consist of whole blood, which includes all blood components: red blood cells (RBCs), white blood cells (WBCs), and platelets suspended in plasma.^35^ During forensic investigations, these samples are collected in their entirety to extract and analyze DNA profiles, identify the source, or determine the presence of illicit substances. In contrast, clinical blood samples often undergo separation into components such as plasma or serum, which alters the protein composition of the sample. Additionally, clinical samples are often stored in tubes coated with ethylenediaminetetraacetic acid (EDTA), an anticoagulant that helps stabilize the molecular components in the blood.^36^ While EDTA is commonly used in experimental studies, its use in forensic blood samples remains questionable, primarily due to its impact on storage, which could significantly affect insights into areas such as the estimation of time since deposition (TSD).^37^ As a result, forensic and clinical blood samples differ considerably in both composition and purpose, making protocols tailored to and data obtained from clinical blood samples unsuitable as references for forensic phenotyping of blood.

Although protein biomarkers for sex have been identified from plasma and serum,^38,39^ the same has not been achieved using whole blood. Public repositories such as PRIDE (PRoteomics IDEntifications Database) and Massive are repositories for extensive proteomics data that could aid in biomarker discovery; however, the lack of sufficient metadata often limits reliable curation and usage.^40^ A deep dive into PRIDE (data gathered on 14/06/2023) resulted in only 28 projects with sufficient metadata to trace the body fluid origin. Out of 5113 runs in these projects, 2000 were on blood constituents (1548 on plasma, 6 on serum, 112 on platelets, and 334 on RBCs). Apart from 29 runs on menstrual blood^41^ (ProteomeXchange identifier PXD007234), none of the well-annotated samples involved whole blood runs. Therefore, for this study, whole blood samples from healthy volunteers were collected and their proteomes were analyzed.

### 3.2 Whole Blood Proteomes Resolved by Biological Sex

A total of 3 597 unique sequences (deriving from 1 366 966 PSMs and mapping to 728 unique protein groups), were confidently identified using an untargeted, LC–MS/MS-based approach. The proteins identified were all known in the PeptideAtlas^42^ Human Plasma 2023–04 build and spanned mostly the upper half of estimated abundances (Figure S1). Of those sequences, 1 711 were confidently quantified, and after filtering for contaminant proteins and missingness over 25% in either of the sexes, a total of 1 561 peptides deriving from 363 protein groups remained (59 of which held a methionine oxidation and 2 a protein N-terminal acetylation). Quantitative precision and missingness were consistent between male and female samples (Figures S2 and S3).

Filtering based on missingness in either sex, while losing peptides completely/mostly missing in either male or female, was done to improve robustness of the results as missingness can have many causes and therefore does not imply absence of a peptide.^43^ Accordingly, peptides with high missingness in either sex would introduce uncertainty, which has to be avoided in the context of forensics. Regardless, no major differences in missingness between male and female samples were noted (Figure S3), likely due to identification transfer during quantification, and no proteins encoded on the Y chromosome (which would be sex-specific by definition) were confidently quantified in the current study. All blank runs were processed in the same way and yielded no quantifiable peptides, showing limited carry-over.

The training set was initially evaluated at the protein level to explore biological sex-related differences. The effect of biological sex on the blood proteome was assessed using a multidimensional scaling (MDS) analysis, which visualizes the similarities between samples (Figure 1 A). The second leading dimension, accounting for 8.03% of explained variance, already somewhat embedded a difference between male and female samples. Despite this apparent separation, the high degree of overlap between male and female samples suggests that a multitude of factors, both technical (e.g., sample loading) and biological (e.g., age, underlying health conditions, and lifestyle), contribute to proteome variability.

**Figure 1:**
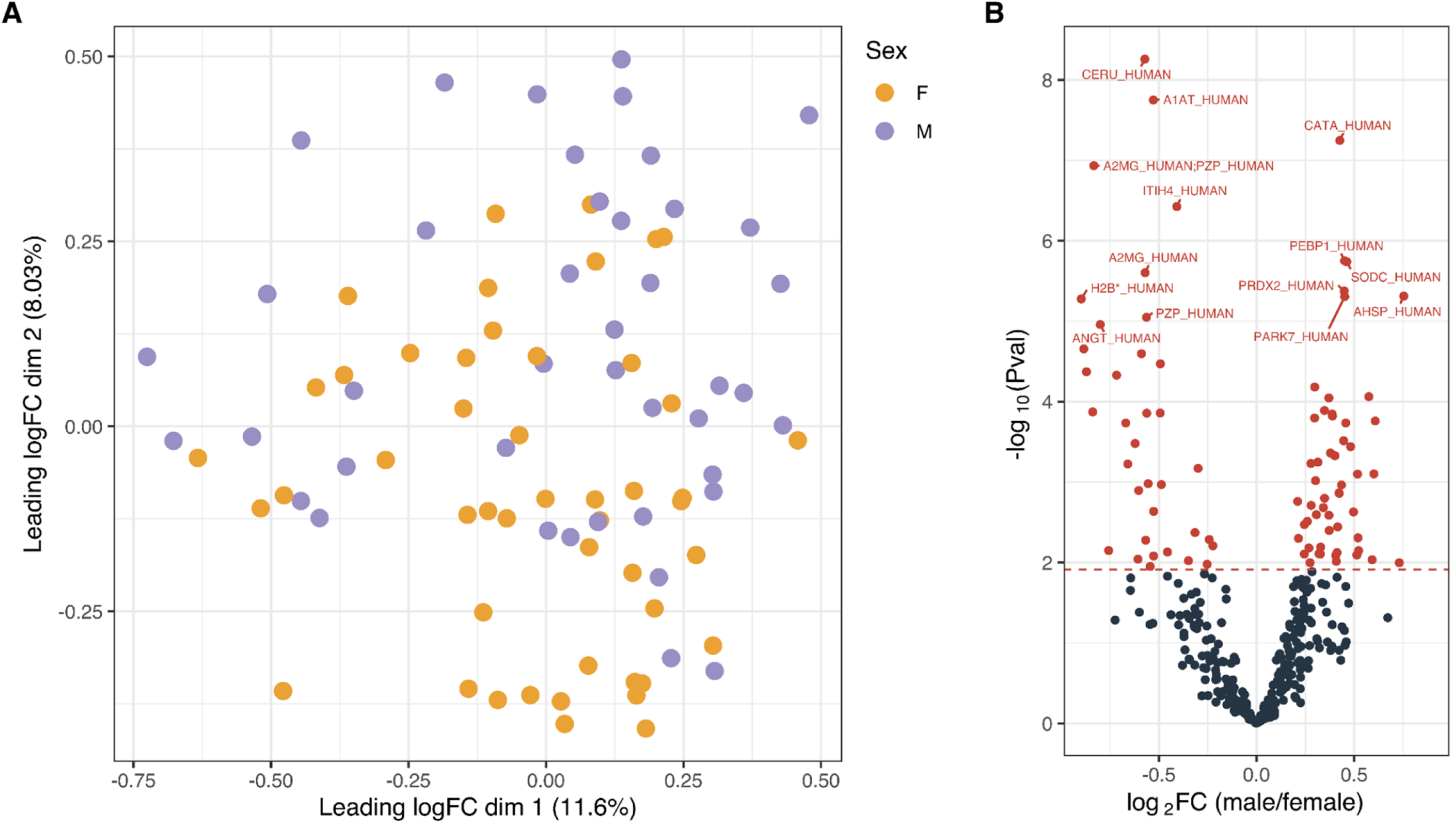
Protein-level analysis reveals distinctions between male and female blood proteomes. (A) MDS plot of blood proteomes showing the approximated typical log_2_ fold change between protein abundance profiles, colored by sex (*F* = female, orange; *M* = male, purple). (B) Volcano plot from differential abundance analysis of proteins. Positive log_2_ fold changes indicate higher abundance in male samples and red dots represent the proteins that are differentiable at an adjusted p-value <0.05 following Benjamini–Hochberg correction.

Additionally, differential abundance analysis between male and female whole blood samples revealed 88 protein groups with significant differences (Figure 1 B). Although these differences were statistically significant in a cohort of 100 donors, the magnitude of change was relatively small; for instance, Ceruloplasmin (CP), the most significantly differentially abundant protein, showed a log_2_ fold change of only −0.57. Importantly, these sex-specific differences were compared to publicly available clinical plasma data^44^ (Figure S4). Excitingly, this stringently controlled public data was largely in accordance with the current findings when it comes to sex differences in the proteome, despite the differences in sample matrix (including cellular proteins in whole blood), preparation, and preprocessing. Of note, Alpha-2-macroglobulin (A2M) was found to be significantly differential in both studies yet in disagreement between them. While the literature points toward a (small) increase in A2M concentration from female to male,^45,46^ in accordance with our findings, this exercise shows the value of public data and how this comparison can reveal markers that should receive extra attention.

While these conventional analyses support the idea that whole blood proteomes can provide information on biological sex, and more broadly on the phenotype, they do not inform on predictive value: differential abundance does not consider the multivariate relationship between different peptides (e.g., synergistic effects in classification) and colinearity typical for high dimensional proteomics data, and would be highly prone to overfitting to the current experimental conditions. Therefore, accurately distinguishing between male and female samples solely based on their blood proteomes will likely require a complex model that includes higher-order interactions.

### 3.3 Biological Sex Classification through Proteomics

ML approaches are well suited for identifying robust patterns and extracting meaningful biological information from highly complex data sets.^47^ For readers interested in further background, we recommend the manuscript on exploration of ML for biomarker discovery from proteomics and omics data,^22^ as a useful introduction to machine learning in proteomics and for clarification of the terms used below.

In this exploratory study, we attempted to classify male and female samples directly from peptides quantified in blood using a regularizing gradient boosting approach.^33^ We chose this model as it tends to show high performance for tabular data while allowing for very simple to highly complex models depending on the chosen hyperparameters. Moreover, the inherent ability to handle missingness and to do both L1 (analogous to lasso regression) and L2 (analogous to ridge regression) regularization was well suited to the high-dimensional proteomics data set.

Peptide abundances were preferred as even though using inferred protein abundances can increase power and biological interpretability in an untargeted proteomics experiment, it can also lead to ambiguity in the form of protein groups (i.e., groups of proteins sharing the same peptides). Moreover, it can diminish the effect of differentially modified peptides due to weighted summarization^48,49^ Finally, routine applications of proteomics often rely on targeted assays, which restricts the number of peptide targets per assay to ensure optimal analytical performance, making the selection of only key peptides crucial. That said, variations at the peptidoform level (e.g., PTMs that change after deposition, genetic variations) could compromise the robustness of peptide targets. Redundancy could be built into the classifier to mitigate this risk, for example by including as many predictive peptide features as possible or by targeting different peptides from the most important proteins in follow-up experiments. The need for such redundancy has to be assessed when validating the classifier.

Cross-validation during training allowed for a best-case evaluation of classifier performance where no unforeseen batch effects were present. Here, out of 90 samples, 87.5% of female and 90.5% of male samples were correctly classified (Figure 2A). Most of the correct classifications were assigned a high probability for the true label, whereas incorrect classifications generally showed more uncertainty in their prediction (Figure 2B). Still, some high probability yet incorrect predictions were made. Such misclassification could not be linked to any donor metadata (e.g., BMI, age, or smoking history) or any other technical factors (e.g., sample preparation batches or feature distributions). Regardless, in a typical forensic sample, these factors are usually unknown and thus impossible to account for during classification.

**Figure 2:**
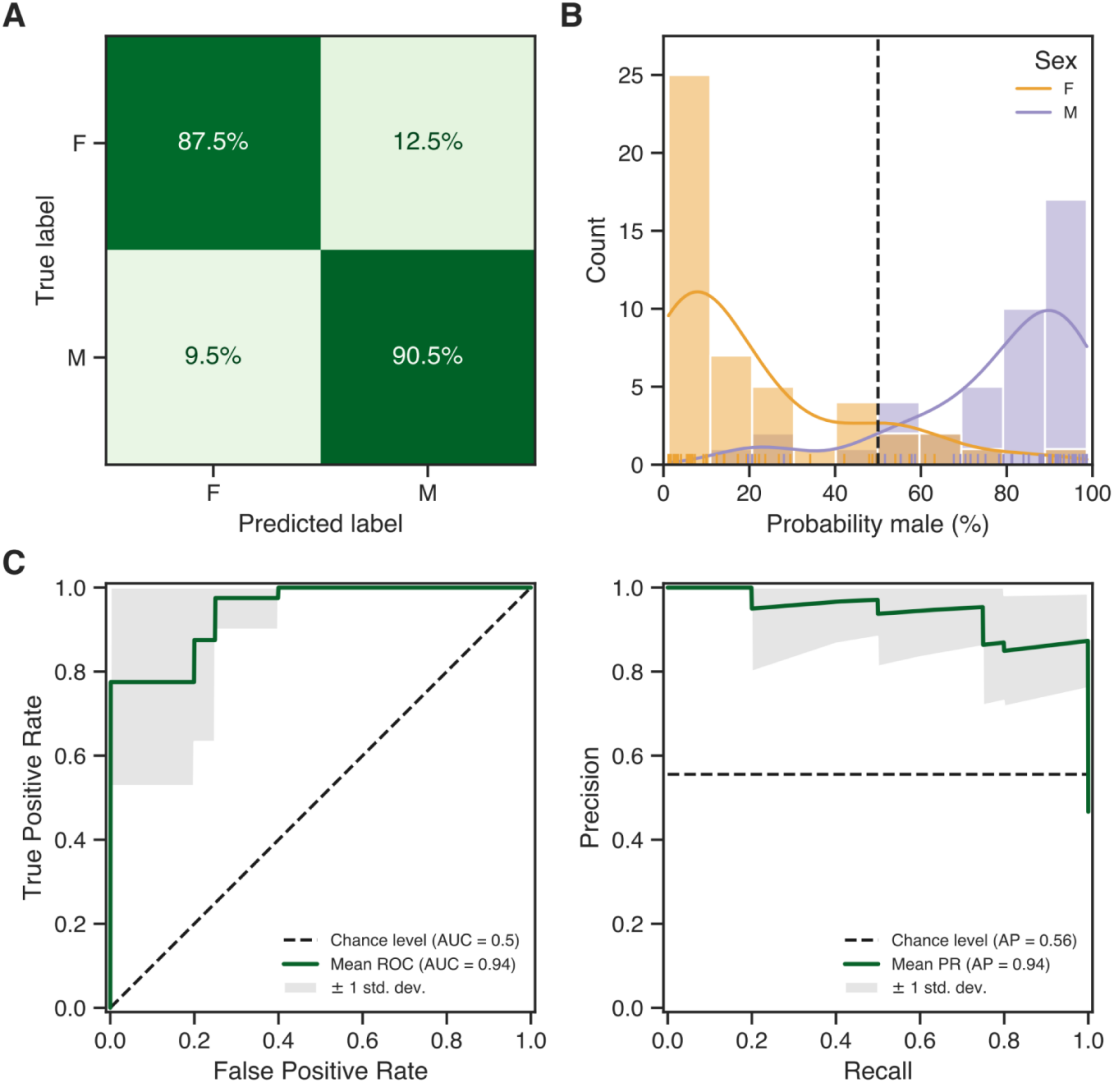
Evaluation of classifier performance through outer cross-validation. (A) Confusion matrix showing the percentage of predicted labels that match (or do not match) their true labels. (B) Probability distribution of sex predictions, colored by sex: female in orange, male in purple. A probability of 0% indicates certainty for females, 100% indicates certainty for males, and 50% represents the threshold of uncertainty between males and females. (C) Mean ROC curve (left) and precision–recall curve (right) over the 10-fold outer split (AUC = area under the curve, AP = average precision).

Instead, misclassification could stem from innate interindividual variations that obscure the difference between male and female blood proteomes. A model trained on a larger cohort and on higher quality data, such as from a targeted assay, should be able to better discern such cases, reducing misclassifications and increasing prediction confidence. While costly and not trivial, increasing sample size will be a necessity to achieve the best possible performance before proceeding to rigorous validation. Nevertheless, the area under the receiver operating characteristic (ROC) curve and average precision were both high, indicating strong classifier performance (Figure 2C). Although these values are somewhat unreliable due to the limited size of the data set, the consistent performance across multiple cross-validation splits demonstrates the potential of the classifier for classifying whole blood samples into biological males and females based on their proteomes.

### 3.4 Classifier Explanations Lead to Target Peptides

Given the exploratory nature of this work, the classifier was trained on untargeted proteomics data. Here, DDA was chosen to allow for the possibility of differentially modified peptidoforms (e.g., PTMs or *in vivo/ex-vivo* cleavage resulting in semi- or nontryptic peptides,^50,51^ ). DDA targets individual precursor ions in contrast to DIA’s highly multiplexed data, allowing for more comprehensive identification of unexpected modifications. DDA data is, however, inherently stochastic and therefore unsuitable as input to a model intended for practical, real-world applications. Instead, a set of predefined target peptides that can be consistently measured with optimal analytical performance will be required, typically in the form of an MRM (multiple reaction monitoring) or PRM (parallel reaction monitoring) assay. The transition toward such a targeted assay is more straightforward from data-independent acquisition (DIA) data; moreover, DIA offers less missing data, improved detection of low-abundance peptides, and improved quantification.^52^ Similar research should therefore always consider if differential modification, which might be an inherently less robust marker, is of interest and if DIA should not be preferred instead. Regardless of this choice, the process of determining target peptides would remain the same.

To determine the target peptides and to ensure classifier performance, a *true to the data* interpretation of the classifier that was trained on the entire training data set was conducted using SHAP.^53^ From an initial input of 1561 peptides, a total of 123 peptides was found to contribute to the model output (Figure 3A and Supplementary Table 1). As expected, the peptide abundances of the most important peptides differed significantly between male and female samples (Figure S5). Among these, one tryptic peptide—VVVQTESGGR (Figure S6) from Pregnancy Zone Protein (PZP)—emerged as the most important peptide for decision-making. Notably, 4 out of the other top 10 most important peptides originated from the protein Ceruloplasmin (CP). Here, the inherently collinear nature of peptide-level data leads to splitting of assigned feature importance across peptides originating from the same protein. The reason why CP showed this splitting of importance and not PZP lies in the number of corresponding peptides and their different distributions between male and female samples: CP was assigned multiple differential peptides, whereas PZP had one particularly differential peptide (Figure S7). One possible explanation for this peptide (i.e., VVVQTESGGR) could be differential modification between the sexes, although no mass shifts were identified from this sequence in the open mass search. Understanding the reason for this inconsistent behavior of PZP peptides in follow-up validation studies could reveal the risks associated with this marker and help in optimizing assay robustness. By summing SHAP values to the protein level, a more representative measure of importance could thus be attributed to each potential target (Figure 3B).

**Figure 3:**
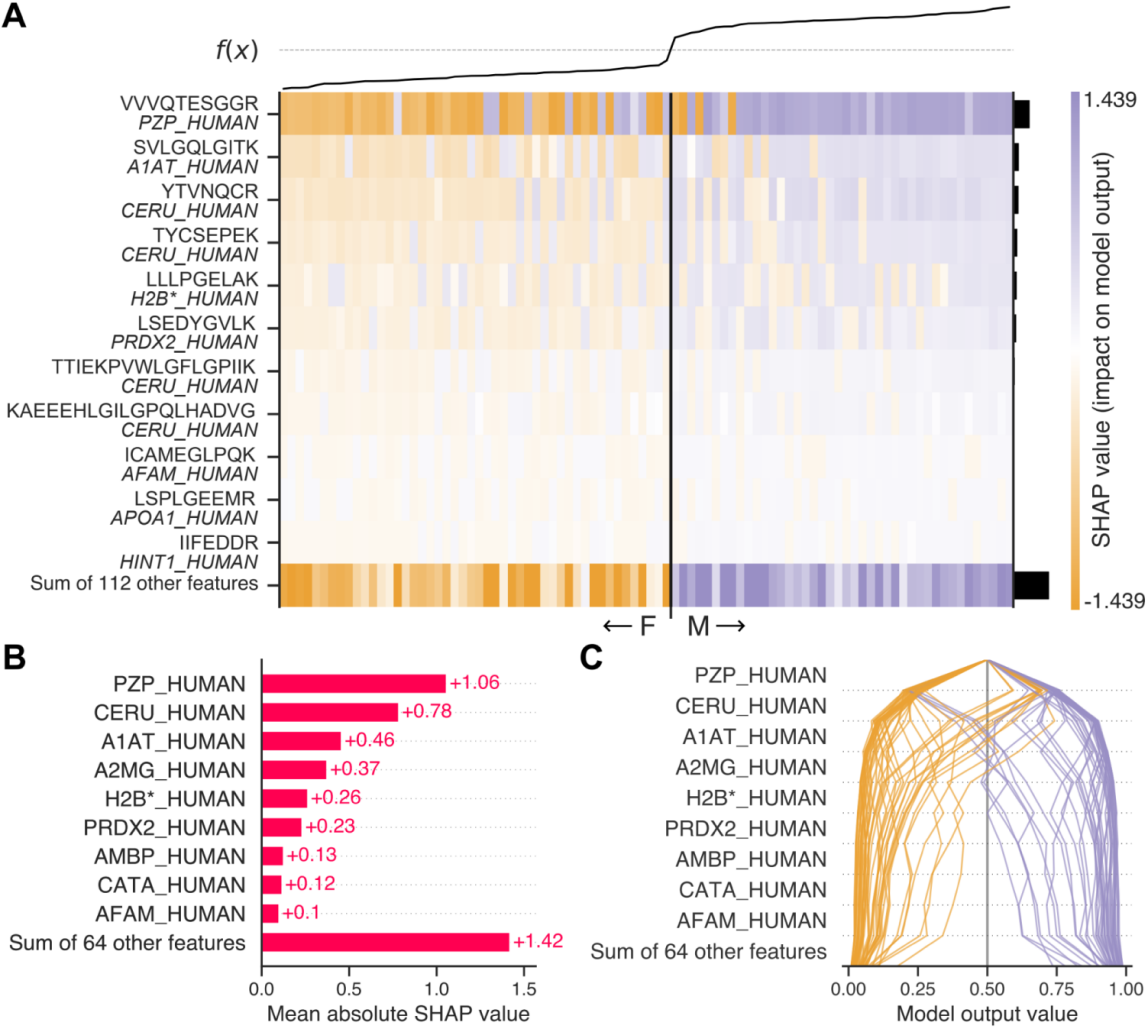
Explaining feature contributions through SHAP values. (A) Heatmap showing the individual feature contributions to the model output for each sample, sorted by increasing probability of a sample being biologically male. Each row corresponds to one peptide feature and its protein of origin, each column represents one sample (B) Mean absolute SHAP values of proteins as a measure of global feature importance, in decreasing order. Protein-level SHAP values were summed from their corresponding peptide-level SHAP values. (C) Decision plot representing a trajectory of each sample to the final model output (i.e., the probability of male sex) in order of decreasing feature importance.

A total of 73 proteins were assigned an importance to the classifier output. The 4 highest importance proteins explained over 50% of the classifier output, and the top 28 proteins together explained over 90%. Some samples were more challenging to classify than others (Figure 3C). To correctly classify all the samples from the training set into biological males and females, the contribution of at least the top 6 most significant proteins was necessary. This signifies the need to measure multiple informative peptides for biological sex classification, in contrast to DNA-based forensic phenotyping. Seeing as targeted assays can be developed for potentially hundreds of peptide targets, all peptides that were assigned an importance (and even additional, redundant peptides of the most important proteins) could be covered in future studies^54,55,56^ . Feature selection during model training could then reduce this number to a more manageable set, reducing cost and complexity while retaining (most of) the model performance.

Protein levels in blood can vary with sex, pregnancy, and contraceptive use, often influenced by hormonal changes. Many proteins expressed in both sexes show higher baseline concentrations in women, with pregnancy causing substantial increases, for example PZP which becomes highly elevated during gestation and IVF but lower in cases of miscarriage^57,58^ Use of contraceptives can also alter protein expression due to hormonal shifts. CP shows sex-based variability, with women expressing higher levels due to elevated copper levels, which can further increase with contraceptive use, especially copper IUDs, through copper ion release,^59,60,61^ . In men, both proteins remain stable and low, highlighting the role of sex hormones in protein regulation. Despite these patterns, the broader effects of hormonal fluctuations, including those from contraceptives, on protein biomarkers remain unexplored in the current study. Further research is needed to clarify how such factors would influence protein expression and diagnostic accuracy to ensure broad applicability of the assay.

Beyond physiological variations, disease states can significantly alter protein levels, including those critical for the classifier. For instance, proteins like alpha-2-macroglobulin and CP are often elevated in conditions such as diabetes, while others, like alpha-1-antitrypsin (A1AT), show altered levels in diseases like gestational diabetes and pre-eclampsia.^62^ In infections like COVID-19, levels of CP and A1AT may be reduced, potentially due to changes in serum copper^63,64^ These alterations emphasize the need for caution when using proteins as diagnostic markers, as the candidate peptide targets could vary significantly, even within an individual. Follow-up studies, validating the robustness of such an assay should include an investigation of the intraindividual variation of the peptide targets.

### 3.5 External Validity and Forensic Adaptability

Most research involving blood samples typically uses collection tubes coated with anticoagulants like EDTA or citrate phosphate dextrose (CPD). In contrast, our study used fresh whole blood that was deposited directly into LoBind tubes (Eppendorf, Hamburg, Germany) containing ABC solution and stored at −20 °C until further use. However, few studies report on the effects of ABC on blood proteins, making it difficult to determine whether ABC preserves the integrity of proteins over time or alters them. At least in part, this could also underly the differences seen in the comparison with the public data depicted in Supplementary Figure 4. Before using specific proteins in forensic contexts, it is therefore crucial to thoroughly understand the impact of ABC or any other storage medium on protein stability.

**Figure 4:**
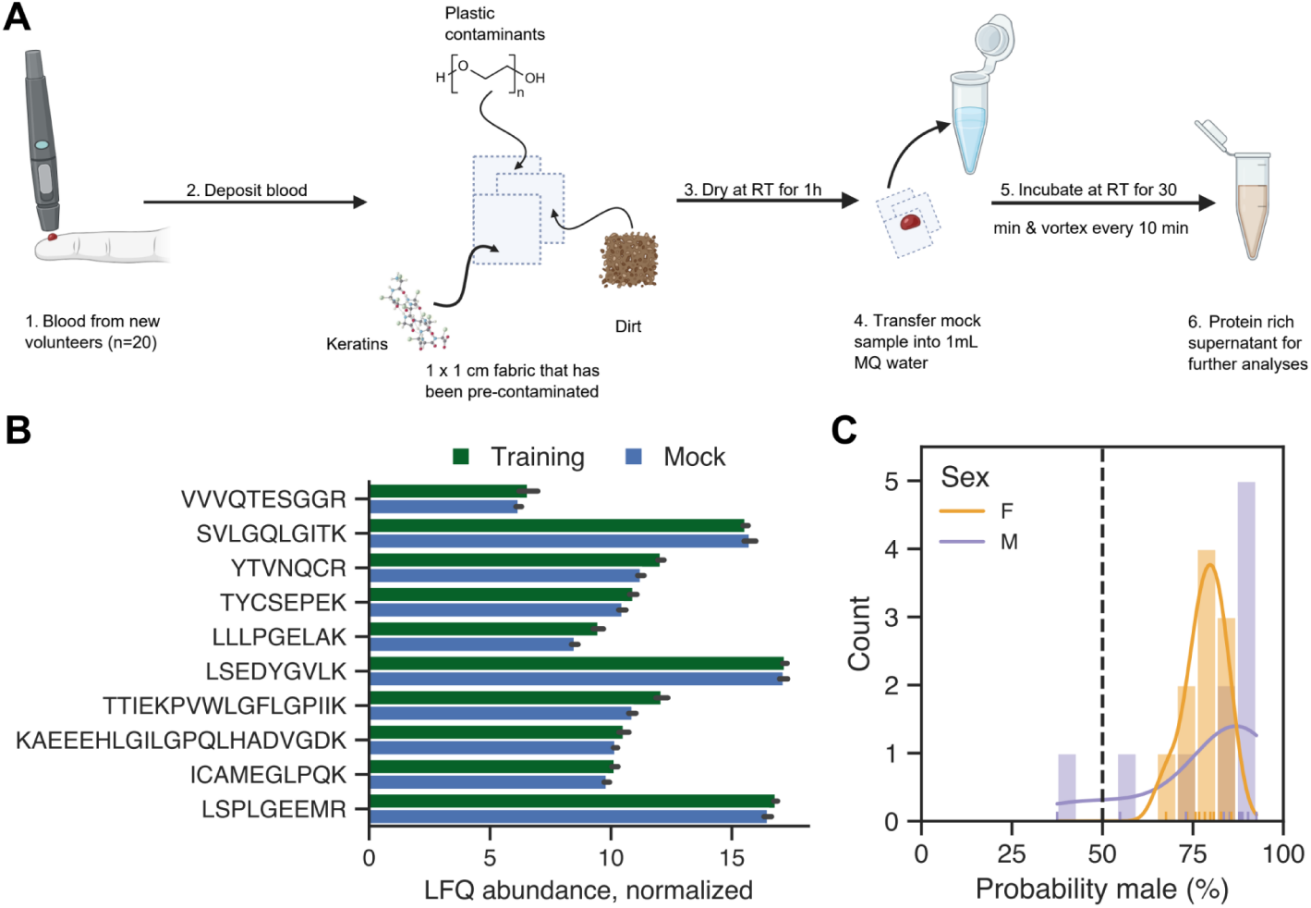
Mock samples more realistically test classifier performance. (A) Mock samples were generated by deposition of whole blood onto substrates that were precontaminated with potential environmental contaminants *(Created with BioR* ender.com/w38h230 *).* (B) Mean log-transformed, normalized peptide abundances of the top 10 most important features within the training and mock data set. (C) Probability distribution of sex predictions from mock samples, colored by sex (*F* = female, orange; *M* = male, purple). A probability of 0% indicates certainty for females, 100% indicates certainty for males, and 50% represents the threshold of uncertainty between males and females.

As previously mentioned, proteomic analyses of plasma and serum have previously identified biomarkers for sex differentiation,^38,44,65^ While valuable information, these sample matrices are not representative of whole blood from forensic trace samples. Moreover, to simplify protocol execution and improve robustness with the aim of routine implementation, depletion or fractionation was omitted from sample preparation in comparison to many plasma and serum studies. In contrast, whole blood proteomics should provide an opportunity to analyze all protein components within a trace sample without introducing any bias.

Additionally, crime scene samples are typically affected by diverse factors including the type of substrate and various environmental conditions like temperature, humidity, and light exposure.^66^ Different types of substrates can induce biochemical changes that affect the drying time with whole blood and plasma each exhibiting distinct drying times and patterns depending on the substrate used^67,68^ Research has shown that higher storage temperatures accelerate the bloodstain aging process, whereas storing them at lower temperatures decelerates the rate of decomposition, helping preserve blood properties over time.^69^ Although not entirely nondestructive, deep freeze storage can effectively prevent postcollection aging, which would otherwise make certain bloodstain properties undetectable. Finally, it is important to consider that crime scene samples are often exposed to outdoor environments, where they can be affected by variations in weather conditions, soil composition, air pollution, and potential microbial growth. A few studies have explored the impact of outdoor environmental conditions, such as wind, rainfall, and humidity, on microRNA (miRNA), mRNA, and DNA^70,71^ However, limited research has been conducted on how these factors affect the (blood) proteome.^72^

To evaluate the potential impact of such factors on the classifier’s performance, mock samples were prepared to simulate the deposition of whole blood on a cotton substrate combined with environmental contamination (Figure 4A). In-house data (*not included in this manuscript*) from real forensic case samples frequently reveal the presence of both proteinaceous and nonproteinaceous contaminants, such as keratins and plastic residues like polyethylene glycol. These contaminants are known to induce matrix effects, which can reduce the detection sensitivity and compromise the reliability of the classification results. A modified sample preparation based on suspension trapping (S-Trap) was employed to remove nonproteinaceous contaminants. While key peptide features could still be quantified from these samples, their average abundance often differed from that of the training set (Figure 4B). This can be partly attributed to the change in sample preparation as varying peptide properties can influence recovery depending on the method used. A more challenging contributing factor is that of batch effects (Figure S8). This arises from technical differences between analytical batches, such as deviations in instrument performance, reagent batches, or environmental conditions. The current model was trained on label-free relative quantification data, which is particularly prone to these batch effects^73,74^ Generally, batch effects can be accounted for or corrected in the case of large batches with uniform sample properties, but it cannot be applied in the forensic context of singular samples with unknown composition.^75^ As such, uncorrected batch effects between training and test samples impacted classifier performance, ultimately leading to failed biological sex prediction of mock samples (Figure 4C). In this case, almost all samples were classified as male due to the abundance of the highest importance peptides being lower on average in the mock set.

Limiting the impact that batch effects have on model performance starts with the implementation of a targeted assay. An MRM/PRM method targets robust transitions at maximum analytical performance thus ensuring high data quality and integrity^76,77^ Additionally, the use of internal standards, such as heavy isotope-labeled peptides, can act as reference points to normalize analyte signals, thereby accounting for and correcting technical variations. Targeted assays are therefore not only essential for routine implementation but also for maintaining robust model performance. As a result, the process of discovery through untargeted approaches such as DDA followed by the development of a targeted assay is already well described^52,78^

With a targeted method fully developed and validated, proper implementation into routine operation is required to further minimize batch effects. Standard operating procedures (SOPs) should be implemented for the entire workflow, from sample collection and preparation up to data acquisition, processing, and analysis. A comprehensive quality control system should include regular checks, maintenance and calibration of MS instruments, consistent monitoring of performance metrics, and the use of quality control samples to detect and measure any deviations that may arise. Once these are in place, a final classification model can be trained and thoroughly validated on a large and high-quality data set generated within the current framework. This would ensure a maximally performant classifier before routine implementation on single samples.

As a final note, in trace investigation and analysis it is essential to preserve the integrity of traces and ensure that no trace is wasted. This makes it crucial to use as little of the trace as possible while maximizing the information obtained. Simultaneous extraction of DNA and proteins from forensic traces has already been demonstrated,^79,80^ a similar compatibility of the current protein extraction protocol with DNA analysis was indicated by the successful recovery of DNA in a theoretically sufficient amount for DNA genotyping (Figure S9). Such a complementary approach should be explored further when incorporating proteomics into the routine forensic toolkit.

## 4 Conclusion

This exploratory study investigated the potential of MS-based proteomics for forensic phenotyping, specifically focusing on the estimation of biological sex. Using an untargeted LC–MS/MS-based proteomics approach, we were able to confidently predict biological sex from whole blood samples. 123 peptides from 73 proteins were found to contribute to the classifier output. Of these, two key proteins (PZP and CP) were found to be most important in distinguishing between biologically male and female samples.

However, we acknowledge there are many challenges to be taken into account in using these proteins for sex classification. The first is the dynamic nature of the proteome, which can be affected by various factors including hormones, contraceptive use, and various health conditions. Rigorous validation of peptide targets across broad demographics and technical circumstances, is required before routine implementation. Therefore, future research should focus on developing an MRM assay for a comprehensive set of sex markers, enabling application to larger and more diverse data sets. This approach would help identify targets suitable for real-world samples and enhance the robustness of the classifier. The estimation of real-world performance is further complicated by two factors: the relevance of the samples and batch effects. The former was addressed through the generation of mock samples, simulating substrate deposition and environmental contamination, although this only serves as a basic approximation of real forensic conditions. The latter will involve transitioning to a maximally analytical targeted assay.

Ultimately, while this study demonstrates some potential of proteomics in forensic phenotyping, the successful implementation of such approaches is highly challenging, requiring careful consideration of the dynamic nature of the proteome and the technical implications of routine proteomics analysis. Before any future research on forensic proteomics phenotyping, these challenges should be weighed against the proposed gains. Nevertheless, proteomics remains a powerful tool in its role as a complementary method supporting investigations where traditional analyses fall short. As such, we believe proteomics might hold greater potential for other applications in forensics, such as source attribution and event reconstruction.

## 5 Declaration

During the preparation of this work, the author(s) used ChatGPT 4.0, an artificial intelligence developed by OpenAI, to assist in their redaction. After using this tool/service, the author(s) reviewed and edited the content as needed and take(s) full responsibility for the content of the publication.

## Supplementary Material





## Data Availability

The MS proteomics data has been deposited to the ProteomeXchange Consortium via the PRIDE (https://www.ebi.ac.uk/pride/)^81^ partner repository with the dataset identifier PXD056606. All code related to the current manuscript is available in the GitHub repository at https://github.com/rualmey/forensic-blood-proteomics.

## Notes

Supplementary Figure S1. Signal and estimated abundance range. Supplementary Figure S2. Protein abundance coefficients of variation in the three experimental data sets. Supplementary Figure S3. Data completeness of the three experimental data sets. Supplementary Figure S4. Correlation between experimental and public data. Supplementary Figure S5. Abundances of the most important peptide features. Supplementary Figure S6. Spectral match of the most important peptide VVVQTESGGR. Supplementary Figure S7. Abundances of peptides from the most important proteins. Supplementary Figure S8. Classifier performance on the test set. Supplementary Figure S9. DNA recovery after the proteomics workflow (PDF)
Supplementary Table 1. SHAP values and Ranks for proteins and peptides (ZIP)
