# Supplementary material for: Potential of Proteomics in Forensic Phenotyping: A Focus on Biological Sex Estimation
Source: J Proteome Res. 2025 Sep 3;24(10):4888–99. doi: 10.1021/acs.jproteome.5c00098 (PMC12501993; doi:10.1021/acs.jproteome.5c00098)
Supplement: Supplementary file 1 [file pr5c00098_si_001.pdf]

Supplementary Information

Title: Potential of Proteomics in Forensic Phenotyping: A Focus on Biological Sex Estimation

Reference ID: pr-2025-000983

Shirin Alex [1 †], Ruben Almey [2 †], Rachel Sian Dennis [2], Olivier Tytgat [2], Robbin Bouwmeester [3, 4], Dieter Deforce [2], Marcel De Puit [1, 5], Maarten Dhaenens [2 * §], Laura De Clerck [2 §]

[1] *Netherlands Forensic Institute, Laan van Ypenburg 6, Den Haag, The Netherlands*

[2] *ProGenTomics, Faculty of Pharmaceutical Sciences, Ghent University, Ottergemsesteenweg 460, 9000 Ghent, Belgium*

[3] *VIB-UGhent Center for Medical Biotechnology, Technologiepark-Zwijnaarde 75, 9052 Ghent, Belgium*

[4] *Department of Biomolecular Medicine, Ghent University, Corneel Heymanslaan 10, 9000 Ghent Belgium.*

[5] *Delft University of Technology, Faculty of Applied Sciences, Department of Chemical Engineering, Van der Maasweg 9, 2629 HZ, Delft, The Netherlands*

[†] *S.A and R.A contributed equally to this work*

[§] *M.D. and L.D.C. contributed equally to this work*

[*] Corresponding Author: *Maarten.Dhaenens@UGent.be*

Table of Contents

SUPPLEMENTARY FIGURE S1. SIGNAL AND ESTIMATED ABUNDANCE RANGE. 3

SUPPLEMENTARY FIGURE S2. PROTEIN ABUNDANCE COEFFICIENTS OF VARIATION IN THE THREE EXPERIMENTAL DATASETS. 4

SUPPLEMENTARY FIGURE S3. DATA COMPLETENESS OF THE THREE EXPERIMENTAL DATASETS. 5

SUPPLEMENTARY FIGURE S4. CORRELATION BETWEEN EXPERIMENTAL AND PUBLIC DATA. 6

SUPPLEMENTARY FIGURE S5. ABUNDANCES OF THE MOST IMPORTANT PEPTIDE FEATURES. 7

SUPPLEMENTARY FIGURE S6. SPECTRAL MATCH OF THE MOST IMPORTANT PEPTIDE VVVQTESGGR. 8

SUPPLEMENTARY FIGURE S7. ABUNDANCES OF PEPTIDES FROM THE MOST IMPORTANT PROTEINS. 9

SUPPLEMENTARY FIGURE S8. CLASSIFIER PERFORMANCE ON THE TEST SET. 10

SUPPLEMENTARY FIGURE S9. DNA RECOVERY AFTER THE PROTEOMICS WORKFLOW. 11

SUPPLEMENTARY TABLE 1. SHAP values and Ranks for proteins and peptides (separate file)

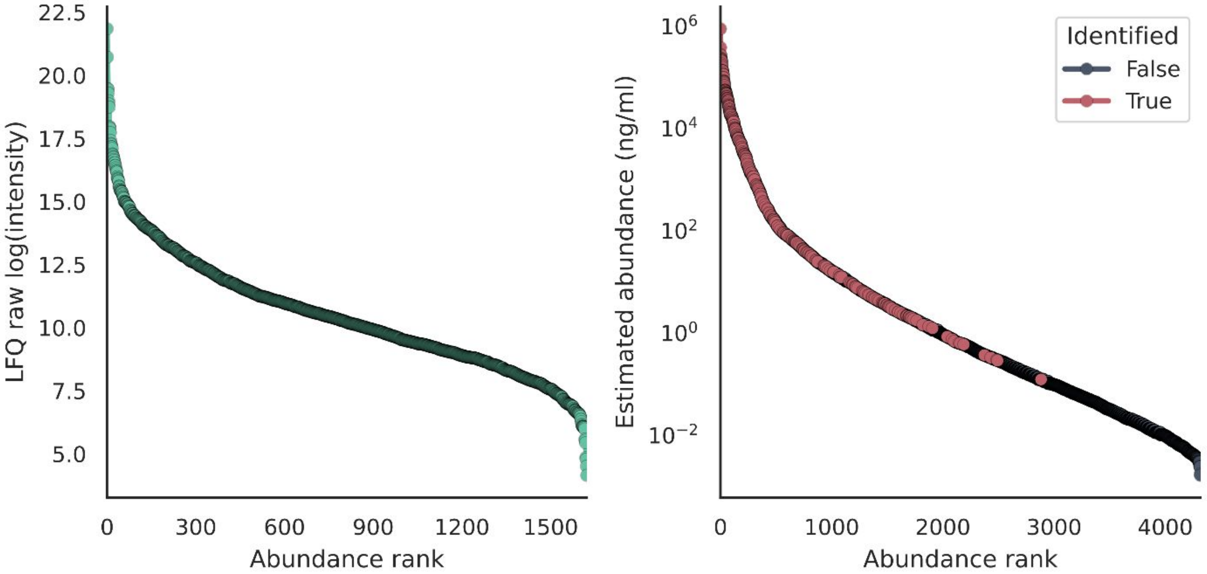

Supplementary Figure S1. Signal and estimated abundance range.

Abundance ranks plots of (left) raw peptide intensities as quantified in the current study, and (right) estimated protein abundances from the PeptideAtlas Human Plasma 2023-04 build, canonical only, with proteins confidently identified in the current study marked in red.

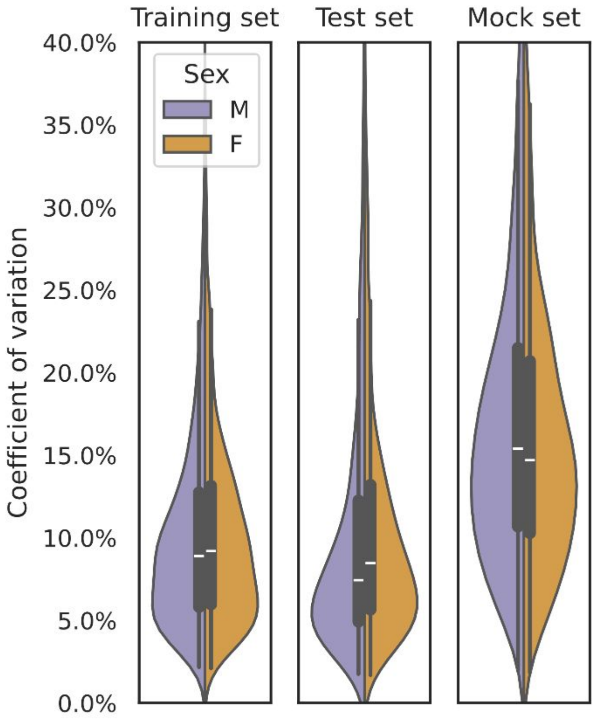

Supplementary Figure S2. Protein abundance coefficients of variation in the three experimental datasets.

Distribution of peptide abundance coefficients of variation of the three confidently quantified peptide datasets, split by sex.

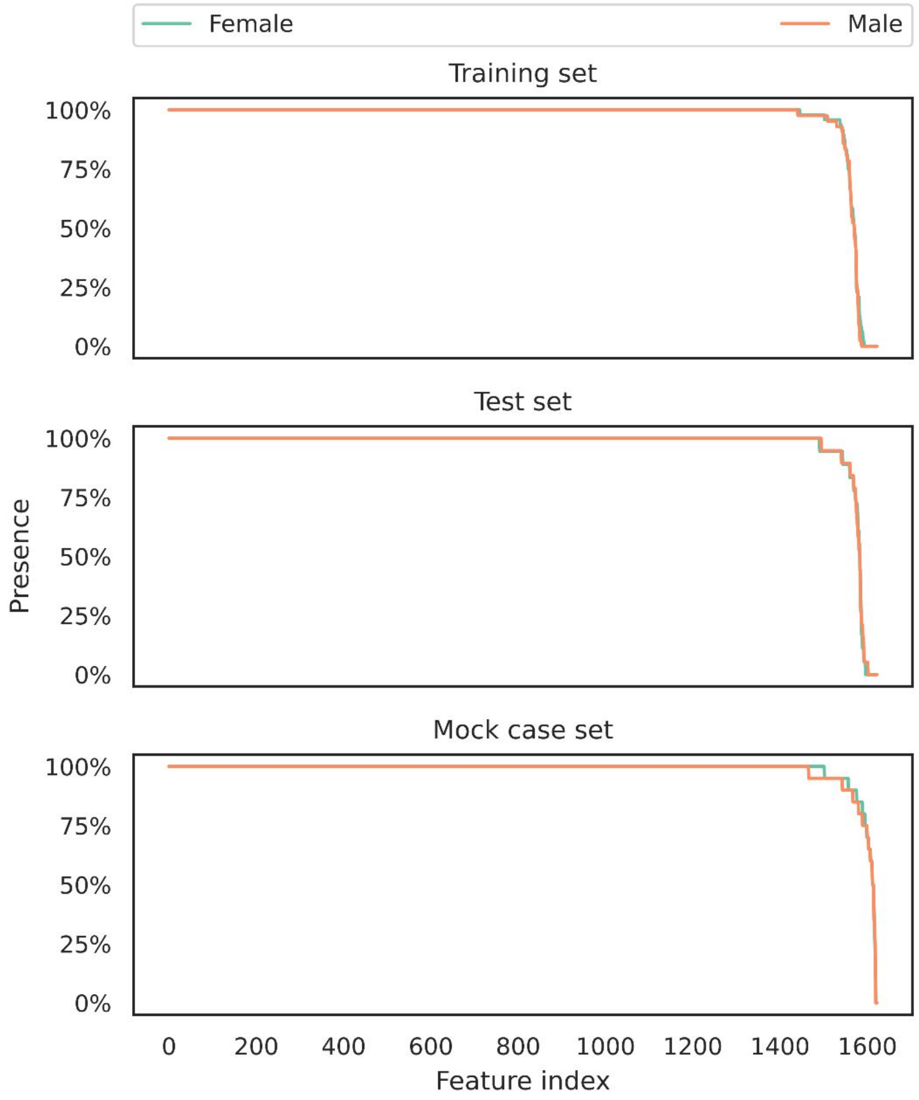

Supplementary Figure S3. Data completeness of the three experimental datasets.

Data completeness of the three confidently quantified peptide datasets, split by sex, and ordered by increasing NA content along the X-axis.

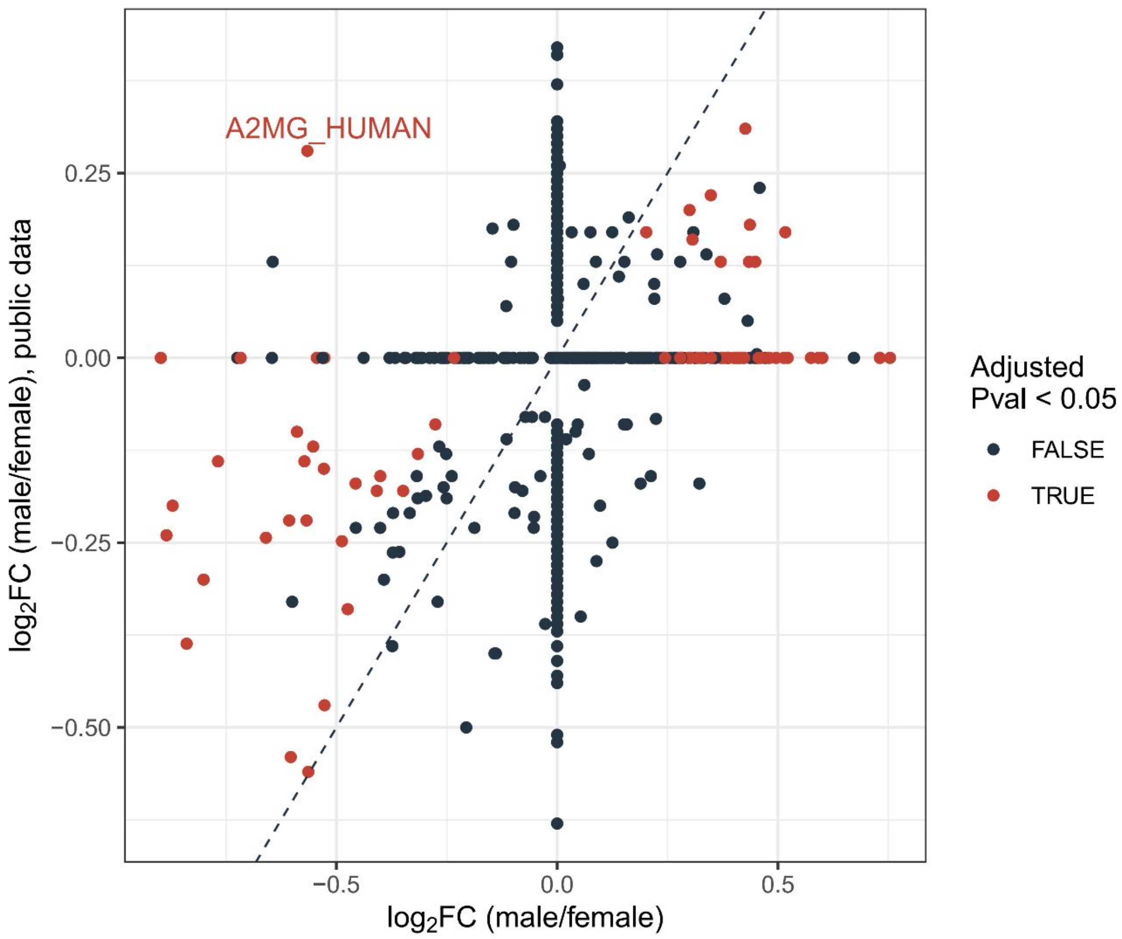

Supplementary Figure S4. Correlation between experimental and public data.

Correlation of protein abundance fold changes after differential abundance analysis between the current experimental data (X-axis) and publicly available data from Niu et al.[44] Only fold changes that were significant were available from the public data, and fold changes that were significant in the current dataset were marked in red.

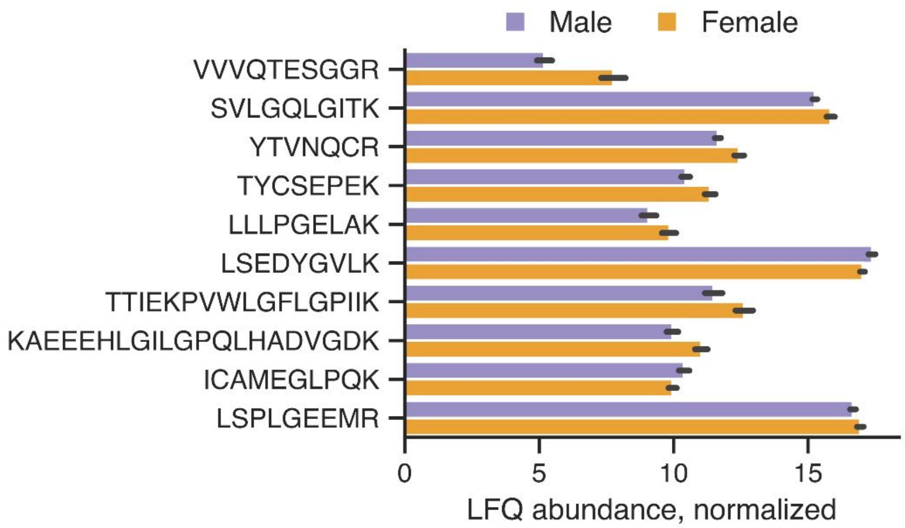

Supplementary Figure S5. Abundances of the most important peptide features.

Mean log-transformed, normalized peptide abundances of the top 10 most important features within the training dataset, by sex

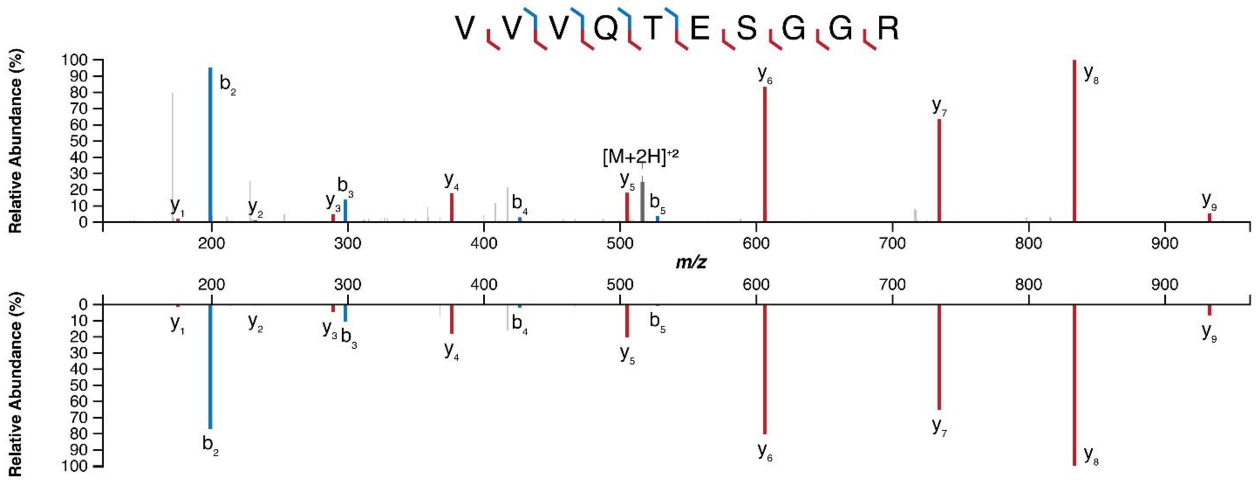

Supplementary Figure S6. Spectral match of the most important peptide VVVQTESGGR.

Experimental spectrum of the highest contributing peptide feature VVVQTESGGR (top) and its corresponding predicted spectrum using Prosit HLA model/HCD (2020) at collision energy 30 (bottom), with b- and y-ions annotated at 25 ppm in blue and red, respectively.

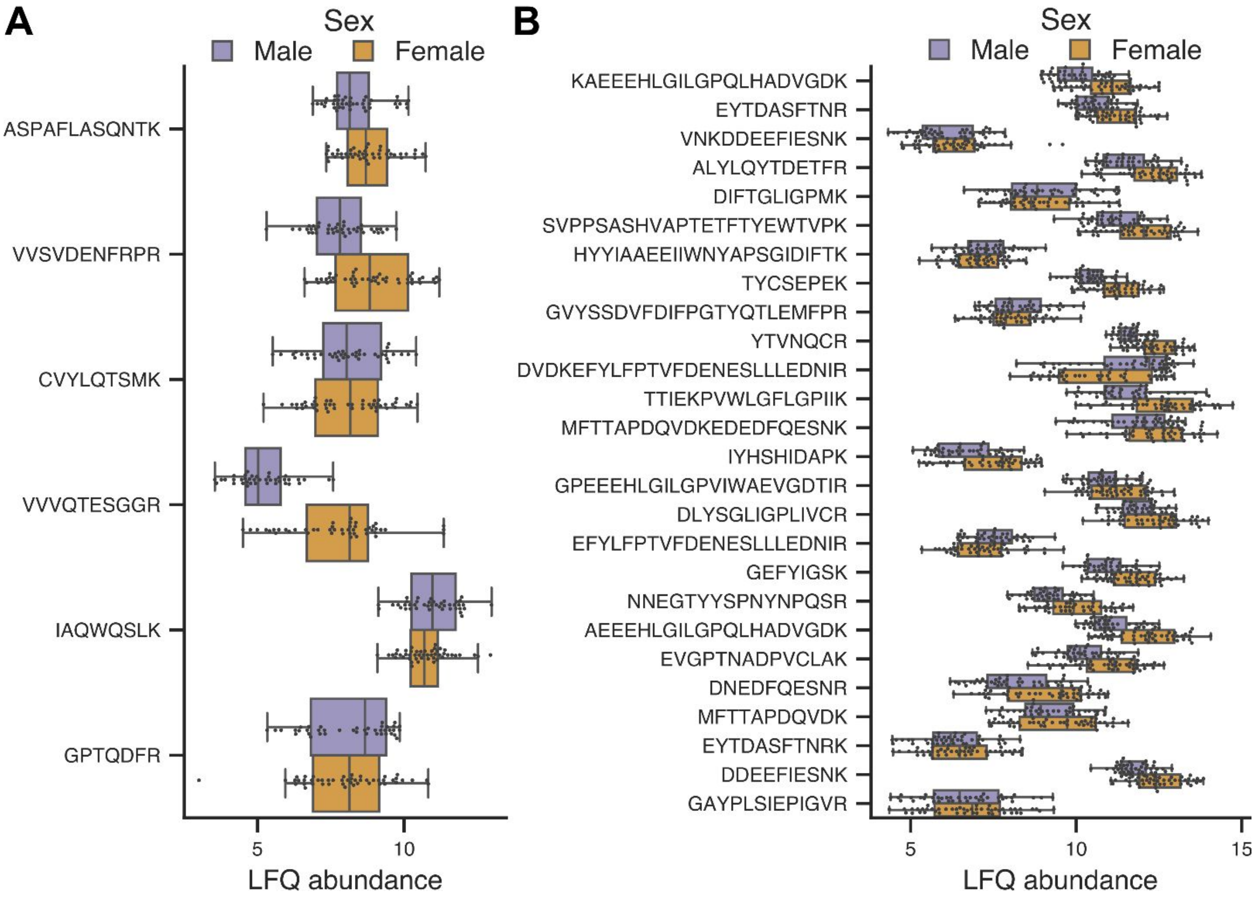

Supplementary Figure S7. Abundances of peptides from the most important proteins.

Normalized abundances of all quantified (A) PZP peptides and (B) CP peptides stratified by sex (female = orange, male = purple).

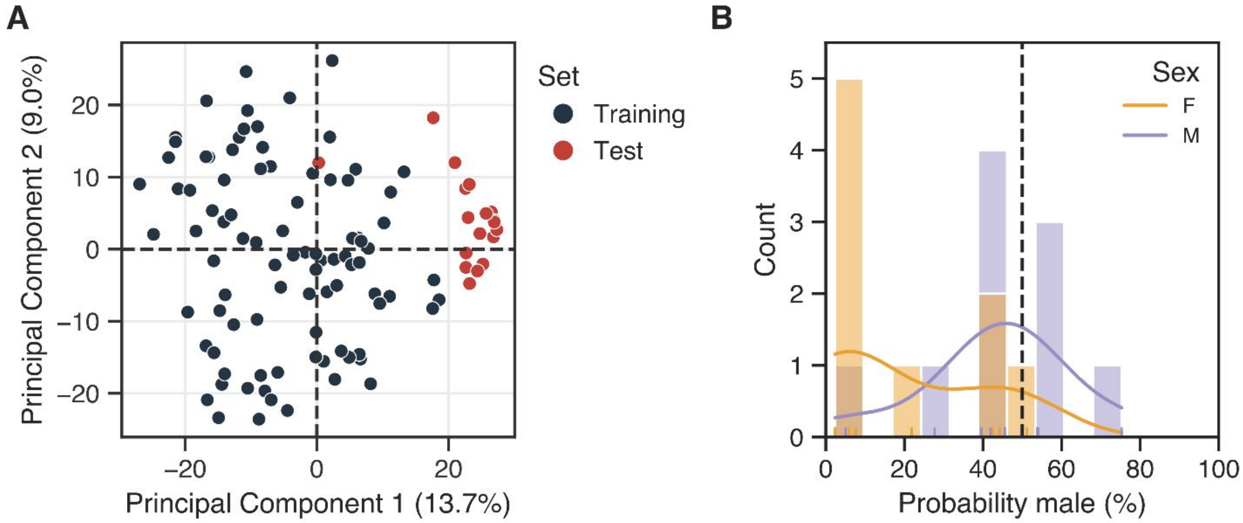

Supplementary Figure S8. Classifier performance on the test set.

Model performance for the test set, i.e., samples that were processed and analyzed in an identical manner as the training set but independently and at a different time. (A) Principal component analysis plot of blood proteomes colored by dataset. Test samples, in red, separate from the training samples along the first principal component. (B) Probability distribution of sex predictions from test samples, colored by sex (F = female, orange; M = male, purple). A probability of 0% indicates certainty for females, 100% indicates certainty for males, and 50% represents the threshold of uncertainty between males and females.

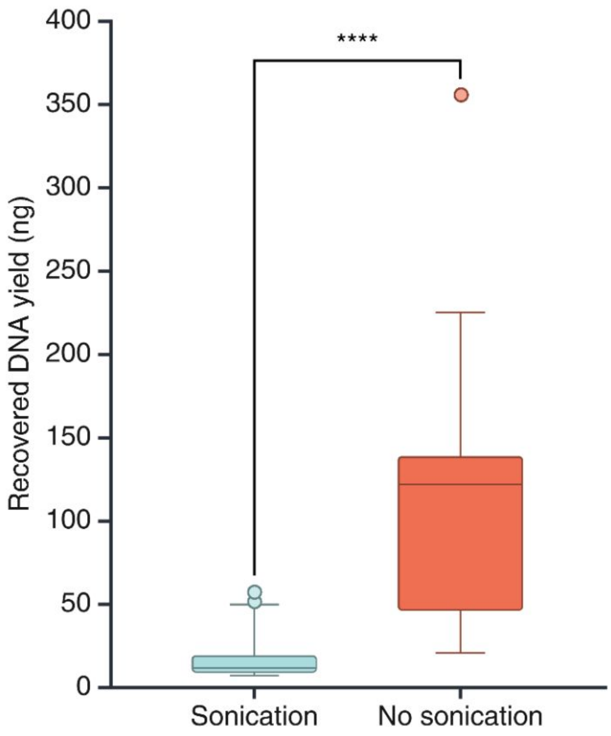

Supplementary Figure S9. DNA recovery after the proteomics workflow.

DNA recovery from the test sample pellets (n = 20) after the first centrifugation (Section 2.2), with or without a preceding sonication step. After centrifugation, 200 µL of Chelex suspension was added to the pellet, followed by thorough vortexing. Samples were incubated at 56 °C for 30 minutes, followed by incubation at 99 °C for 8 minutes. After a final centrifugation at 14,000 x g for 3 minutes, the clear supernatant was stored at -20 °C. Extracted DNA was quantified using the Investigator Quantiplex Kit (Qiagen, Hilden, Germany), according to the manufacturer's instructions. The total reaction volume was 20 µL, using 2 µL of extracted DNA as input. The samples were quantified on a LightCycler 480 II device (Roche, Basel, Switzerland). Thermal cycling consisted of 3 minutes at 95 °C, followed by 40 cycles of 5 seconds at 95 °C and 35 seconds at 60 °C. Determination of the quantification cycle (Cq) was performed by the LightCycler 480 software. The recovered DNA yield was finally calculated for the entire sample (200 µL). Created with BioRender Graph.
